# Does Aerobic Exercise Influence Intrinsic Brain Activity? An Aerobic Exercise Intervention among Healthy Old Adults

**DOI:** 10.3389/fnagi.2017.00267

**Published:** 2017-08-11

**Authors:** Pär Flodin, Lars S. Jonasson, Katrin Riklund, Lars Nyberg, C. J. Boraxbekk

**Affiliations:** ^1^Center for Demographic and Aging Research, Umeå University Umeå, Sweden; ^2^Umeå Center for Functional Brain Imaging, Umeå University Umeå, Sweden; ^3^Diagnostic Radiology, Department of Radiation Sciences, Umeå University Umeå, Sweden; ^4^Physiology, Department of Integrative Medical Biology, Umeå University Umeå, Sweden; ^5^Danish Research Centre for Magnetic Resonance, Centre for Functional and Diagnostic Imaging and Research, Copenhagen University Hospital Hvidovre Copenhagen, Denmark

**Keywords:** aerobic exercise, brain plasticity, aging, fMRI, resting-state, ASL

## Abstract

Previous studies have indicated that aerobic exercise could reduce age related decline in cognition and brain functioning. Here we investigated the effects of aerobic exercise on intrinsic brain activity. Sixty sedentary healthy males and females (64–78 years) were randomized into either an aerobic exercise group or an active control group. Both groups recieved supervised training, 3 days a week for 6 months. Multimodal brain imaging data was acquired before and after the intervention, including 10 min of resting state brain functional magnetic resonance imaging (rs-fMRI) and arterial spin labeling (ASL). Additionally, a comprehensive battery of cognitive tasks assessing, e.g., executive function and episodic memory was administered. Both the aerobic and the control group improved in aerobic capacity (VO_2_-peak) over 6 months, but a significant group by time interaction confirmed that the aerobic group improved more. Contrary to our hypothesis, we did not observe any significant group by time interactions with regard to any measure of intrinsic activity. To further probe putative relationships between fitness and brain activity, we performed *post hoc* analyses disregarding group belongings. At baseline, VO_2_-peak was negativly related to BOLD-signal fluctuations (BOLD_STD_) in mid temporal areas. Over 6 months, improvements in aerobic capacity were associated with decreased connectivity between left hippocampus and contralateral precentral gyrus, and positively to connectivity between right mid-temporal areas and frontal and parietal regions. Independent component analysis identified a VO_2_-related increase in coupling between the default mode network and left orbitofrontal cortex, as well as a decreased connectivity between the sensorimotor network and thalamus. Extensive exploratory data analyses of global efficiency, connectome wide multivariate pattern analysis (connectome-MVPA), as well as ASL, did not reveal any relationships between aerobic fitness and intrinsic brain activity. Moreover, fitness-predicted changes in functional connectivity did not relate to changes in cognition, which is likely due to absent cross-sectional or longitudinal relationships between VO_2_-peak and cognition. We conclude that the aerobic exercise intervention had limited influence on patterns of intrinsic brain activity, although *post hoc* analyses indicated that individual changes in aerobic capacity preferentially influenced mid-temporal brain areas.

## Introduction

Given the increasing disease burden of age related cognitive problems that comes with an aging world population (Christensen et al., 2009), physical exercise could provide a widely available and cost-effective approach to reduce age related cognitive decline at a large scale (Baker et al., 2010). Prospective studies show that low fitness in early adulthood is associated with increased risk for early onset dementia later in life (Nyberg et al., 2014; Wendell et al., 2014). Moreover, cross-sectional population based studies have confirmed that individuals that stay physically active have an improved brain-behavior relationship (Boraxbekk et al., 2016).

Human intervention studies probing the cognitive effects of exercise have, however, showed mixed findings. Meta-analyses have reported a range of effect sizes on exercise induced improvements of cognition among elderly. Smith et al. (2010) reported modest improvements in attention, processing speed (PS), executive function (EF), and memory. Colcombe and Kramer (2003) report medium improvements (of particularly) EFs, whereas a recent systematic meta-analysis concludes that there is no evidence that aerobic exercise benefit cognition among healthy older adults (Young et al., 2015). Thus, there is a need for further investigations of the extent to which physical exercise interventions among elderly could maintain, or even restore, cognitive function and brain health. For investigations of the neurophysiological mechanisms subserving the neuroprotective effects of aerobic exercise, human brain imaging plays a central role (for a review see, e.g., Stillman et al., 2016).

Evidence suggests that resting state brain activity could be sensitive to also early-stage neuroplastic brain changes (Kelly and Castellanos, 2014). To date, very few studies have investigated changes in intrinsic brain activity following a structured physical exercise intervention among healthy older adults. Voss et al. (2010) investigated longitudinal changes in three age sensitive brain networks, using a region of interested (ROI) based seed correlation analysis (SCA), following 6 and 12 months supervised cardiovascular training. Compared to an active control group, the aerobic (walking) group increased connectivity in parts of the default and frontal executive network after 12 months, although no significant group differences were observed at 6 months.

Among the cross-sectional studies linking fitness to intrinsic brain activity among elderly, a commonly reported finding is reversal of age-related changes. Voss et al. (2016) used network based statistics (NBS) on graphs based on hubs defined in age-sensitive networks. These networks were identified as the default mode- (DMN), dorsal attention-, and executive control-, salience- and sensory related networks. Whereas older subjects displayed enhanced between-network connectivity, younger subjects displayed larger within-network connectivity. They concluded that cardiorespiratory fitness among older was positively associated with connectivity within age-sensitive networks, primarily the DMN, whereas no associations were observed for self-reported physical activity. However, another large scale cross-sectional study, Boraxbekk et al. (2016) found that current and accumulated physical activity was associated with stronger integrity of the DMN in the anterior parts of posterior cingulate cortex (PCC).

In the current study, we wanted to expand on the findings relating aerobic fitness to intrinsic brain activity. In addition to investigate resting-state functional connectivity (i.e., correlations of BOLD-signal time series of distributed brain regions), we also examined fluctuations of BOLD-signal time series (BOLD_STD_). Resting state BOLD_STD_ has previously been used as a proxy measure for vascular flexibility (Burzynska et al., 2015). Moreover, vascular stiffness has in previous studies been linked to aging and cognitive decline (Mitchell et al., 2011; Gauthier et al., 2015), and there is evidence that physical activity counteracts age related vascular stiffness. Burzynska et al. (2015) detected a positive relationship between variability in intrinsic brain activity and physical activity (measured with actigraphs), but not for cardiovascular fitness (VO_2_-max). The authors concluded that BOLD-signal fluctuations could provide a putative neural correlate of brain health among elderly, and that “longitudinal and intervention studies will shed more light on the potential of BOLD in detecting changes in brain function as a result of increased physical activity” (Burzynska et al., 2015). Using a more direct MRI based measure of vascular rigidity, aortic pulse wave velocity, Gauthier et al. (2015) reported an increase of vascular rigidity with age, and a negative association to VO_2_-max (i.e., maximum rate of oxygen consumption).

Another way to characterize vascular function (which is a likely target for aerobic exercise) is quantification of cerebral blood flow (CBF). Maass et al. (2015) used gadolinium-based perfusion imaging MRI to investigate longitudinal changes among healthy older adults that partook in a 3-months exercise intervention, and found that improvements in aerobic capacity correlated with increases in hippocampal blood flow. In cross-sectional data, Boraxbekk et al. (2016) found a positive relationship between physical activity and CBF (ASL) in PCC.

To our knowledge, the current study is the first to employ pure resting-state functional magnetic resonance imaging (rs-fMRI) scans before and after an actively controlled, long-term physical exercise intervention among healthy elderly. In the current article, we present the resting-state results of the research project “Physical Influences on Brain in Aging” (PHIBRA), for which the design, cognitive performance, and structural MRI-data recently were reported (Jonasson et al., 2017). By using a comprehensive set of analytical approaches of rs-fMRI data, and additional measures of ASL-CBF, we attempted to replicate and extend on previous research that evaluates the impact of aerobic fitness on intrinsic brain activity. Based on previous studies we predicted that analyses of group by time interactions would reveal differential longitudinal changes in (1) hippocampal resting state functional connectivity and betweenness centrality, (2) DMN integrity (3) voxel wise BOLD_STD_ and CBF. Additionally, we aimed to characterize relationships between rs-fMRI and fitness using more exploratory approaches. These included multivariate representations of whole brain connectivity (in the following referred to as multivariate pattern analysis, connectome-MVPA), NBS and graph theoretical measures (global efficiency). In order to investigate the functional significance of any observed fitness-brain relationship stipulated above, we performed *post hoc* analyses relating neurophysiological changes to changes in cognition.

## Materials and Methods

### Subjects

Sixty healthy but sedentary older adults (age 64–78 years) were recruited and randomized into performing either supervised aerobic exercise training, or stretching and toning control training, for 6 months, three times a week (30–60 min per session). In the present analysis, 13 subjects were excluded due to the following reasons: one subject was excluded due to brain abnormalities; two subjects were unable to complete the intervention; one person was excluded since baseline VO_2_-peak exceeded the group average by three standard deviations. Another nine persons were excluded due to suprathreshold movement during resting state scans at either pre- or post intervention sessions, see criteria below. Thus, complete data were obtained from 47 subjects, 22 in the aerobic intervention arm and 25 in the active control group. The groups did not significantly differ with respect to age [*t*(45) = 0.92, *p* = 0.36], sex [χ2(1) = 0.045 *p* = 0.83], education [*t*(45) = 0.1, *p* = 0.36], or BMI [*t*(45) = 1.01, *p* = 0.32], see **Table 1**.

**Table 1:** Demographics, and longitudinal changes in VO_2_-peak and graph measures.

	Aerobic *n* = 22			Control *n* = 25			Group × time	
Measure	Pre	Post	*G*	Pre	Post	*G*	*F*(1,43)	$\eta_{p}^{2}$
Age	68.41 (2.59)	–	–	69.16 (3.01)	–	–	–	–
Sex (females)	13	–	–	14	–	–	–	–
Education (years)	13.77 (3.74)	–	–	13.84 (5.02)	–	–	–	–
BMI	25.85 (3.52)	–	–	26.92 (3.41)	–	–	–	–
VO_2_ peak	21.37 (3.83)	27.87 (6.07)	**1.26 (0.9**-**61.80)**	19.59 (3.25)	23.56 (5.12)	**0.91 (0.66**-**1.30)**	**6.20 (0.02)**	**0.12 (0.01–0.33)**
Global efficiency	0.20 (0.01)	0.21 (0.02)	**0.39 (0.05**-**0.83)**	0.21 (0.02)	0.21 (0.01)	0.18 (-0.17–0.68)	0.7 (0.39)	0.02 (0.00–0.14)
BetweenCent L hippo	293.45 (238.90)	209.45 (110.79)	–0.44 (-0.97-0.12)	300.40 (336.28)	251.52 (289.14)	–0.15 (-0.66–0.36)	0.45 (0.50)	0.01 (0.00–0.14)
BetweenCent R hippo	182.09 (144.63)	143.09 (176.50)	–0.24 (-0.94-0.24)	127.52 (120.91)	173.68 (203.12)	0.27 (-0.22–0.67)	1.35 (0.25)	0.03 (0.00–0.16)

*Within group mean values (for pre- and post measures) are reported with SD in parenthesis. Effect sizes for within group longitudinal changes are reported as Hedges *G*, with lower and upper bound for 95% CI (bootstrapped, *n* = 10000) in parentheses. Group by time interactions are tested using repeated measure ANOVA, where the *F* statistics are reported with the significance level in parenthesis. Effect sizes are specified using eta-squared, with the lower and upper bound in parentheses. Significant (unc) effects are in bold. Groups did not significantly differ with regard to age [*t*(45) = 0.92, *p* = 0.36] or sex [χ2(1) = 0.045 *p* = 0.83].*

For the ASL analyses, pre- and post intervention data was available from 55 subjects (aerobic group: 30 subjects, 16 females; control group: 25 subjects, 14 females), age 68.7 ± 2.79 years. There were no significant group differences in age, [*t*(53) = -1.27, *p* = 0.21), sex χ2(1) = 0.040 *p* = 0.843], education [*t*(53) = -0.087, *p* = 0.93], or BMI [*t*(53) = -0.98, *p* = 0.33]. For complete sample characteristics, see Jonasson et al. (2017).

### Intervention Procedures and Behavioral Outcome Measures

For detailed descriptions of the intervention, the VO_2_-peak assessment, and the cognitive test battery, see Jonasson et al. (2017). In short, the intervention procedure was as follows: Subjects were recruited from local newspaper advertisement. Prior to randomization into either of the two intervention arms, all subjects underwent pre-intervention testing. The testing consisted of visits to the lab at six different days. On the first testing day participants visited the Sports Science Lab, Umeå University, where fitness measures, including VO_2_-peak, were assessed using a standardized graded cycle ergometer test. Neuropsychological testing was performed on three separate days. Assessments of cognitive ability included three tests designed to tax (verbal) episodic memory (EM), three test for EF, as well as other tests including assessments of working memory updating (UPD) and PS. A composite score, cognitive score (CS), was computed as a unit-weighted average of EM, EF, UPD, and PS, as a single metric of overall cognition. Finally, MRI-data of different modalities was collected, of which the rs-fMRI and the ASL-CBF data is reported here. Additional questionnaires and dopamine positron emission tomography (PET) imaging were also collected, but will be reported elsewhere. The testing was conducted both before and after the interventions. The cognitive testing, including the tests assessing EM, EF, and CS that are used here, is described in detail in Jonasson et al. (2017).

### Acquisition

Brain imaging data was acquired on a 3 T GE scanner equipped with a 32-channel head coil. High-resolution T1-weighted structural images were acquired using the following parameters: 180 slices; 1 mm thickness; repletion time (TR) 8.2 ms; time to echo (TE) 3.2 ms; flip angle 12°; FOV 25 cm × 25 cm. Functional data were acquired with a gradient EPI sequence with the following parameters: 37 transaxial slices, 3.4 mm thickness, 0.5 mm gap, TR 2000 ms, TE 30 ms, flip angle 80°, FOV 25 cm × 25 cm, inplane resolution of 2.6 mm × 2.6 mm. During resting state scan acquisition, subjects were instructed to lie still and keep their eyes on a white fixation cross for 9 min and 40 s, resulting in 290 volumes per subject and session.

Whole brain perfusion was measured with a 3D pseudo-continuous ASL sequence. Labeling time was 1.5 s, post-labeling delay time was 2 s, field of view was 24 cm, slice thickness was 4 mm, number of averages was 3, number of control label pairs was 30, and acquisition resolution was 8 × 512 (arms × data points in spiral scheme). Forty slices covered the whole brain and the reconstructed voxel size was 1.88 mm × 1.88 mm × 4 mm. CBF maps were computed using the standard GE reconstruction, showing tissue CBF in ml/min/100 g.

### MRI Data Preprocessing and Denoising

Rs-fMRI data preprocessing was performed in SPM12 using standard preprocessing steps. These included slice time correction, realignment and unwarping using 6th degree B-spline interpolation, and functional to structural coregistration. Structural images were segmented into gray matter, white matter and cerebral spinal fluid images. Coregistered functional and structural images were normalized to standard space using Geodesic Shooting as implemented in the Shoot toolbox in SPM12 (Ashburner and Friston, 2011). In short, a common anatomical group-average 1 mm isotropic template was generated, and subject specific deformation fields were created and used for warping T1- and T2^∗^-weighted images from subject space to group template space. Secondly, non-linear deformation fields pushing images from group space to MNI space were applied. Functional data was then resampled into 2 mm × 2 × 2 mm and finally smoothed using a Gaussian kernel of 8 mm.

Cerebral blood flow data was coregistered to structural data, and normalized using the same normalization procedures as applied to the structural and functional data.

At the subject level, rs-fMRI time series were denoised by controlling for: (1) suprathreshold movement (frame wise displacement >0.25 mm or >3 std change in global signal intensity, similar to, e.g., (Satterthwaite et al., 2013), (2) signals from white matter and cerebrospinal fluid (five most explanatory principal components from each tissue mask), and (3) six movement parameters obtained from spatial realignment plus their time derivatives. Subsequent to nuisance regression, time signals were filtered (band passing 0.008–0.09 Hz). Nine subjects lost >50% of their volumes either at pre- or post intervention sessions (of originally 290 volumes) due to movement censoring, and were therefore excluded from further analysis. Denoising of data was accomplished with the Conn toolbox (version 15.h).

### fMRI Analysis

Longitudinal effects of the intervention were evaluated as group by time interactions. Voxel-wise group analyses of functional connectivity were performed using the Conn toolbox, where the two experimental groups were compared in terms of longitudinal changes with regard to the resting state metrics. Likewise, 2-sample *t*-tests of the post minus pre intervention BOLD_STD_ and CBF volumes were analyzed using the 2-sample *t*-test as implemented in SPM12. Group level statistics of graph-theoretical indices (of betweenness centrality and global efficiency) were calculated using repeated measure ANOVA (using MatLab) with group and time as factors.

*Post hoc* analyses aimed to link individual longitudinal changes in VO_2_-peak to changes in brain activity were also conducted, ignoring experimental group-belonging. For these we performed linear regressions, where changes in brain activity were regressed on changes in VO_2_-peak. To guide longitudinal analyses, rs-fMRI data were first related to aerobic capacity at baseline. Any obtained significant brain-fitness association at baseline was used to inform subsequent longitudinal analysis (see below).

In all group analyses, we controlled for age and sex (as e.g., done in Maass et al., 2015). For the longitudinal analyses, we also controlled for baseline ratings of VO_2_-capacity. Additionally, since the two experiential groups were matched with regard to sex and age, the analyses of group by time interactions were also performed without control for sex, age and VO_2_ – peak ratings. In all second-level fMRI analyses, we controlled for mean frame wise displacement (FD).

#### Seed Based Correlation Analysis

Voxel-wise seed based correlation analyses (SCA) were performed using the Conn toolbox (15.h). For each subject and each seed region, *z*-transformed Pearson correlation maps were brought to second level group analysis. We defined a priori seeds in brain regions that in previous literature have been influenced by aerobic exercise. These included (a) left and right hippocampus obtained from the SPM Wake Forest University (WFU) Pickatlas toolbox (Maldjian et al., 2003), (b) left and right parahippocampus (WFU pickatlas), and (c) PCC, based on a cluster reported by Boraxbekk et al. (2016) (center of gravity MNI coordinates -6, -32, 27; 67 voxels).

#### BOLD-Signal Fluctuations

Variations in the preprocessed and cleaned resting state BOLD time series were quantified using FSL maths^1^, rendering one voxel-wise whole brain map of the standard deviations (BOLD_STD_) for each subject and each session. As a corroborating measure, we also calculated Amplitude of Low Frequency Fluctuations (ALFF) which quantifies the total power within the lower frequency band. ALFF was calculated for the same preprocessed and cleaned volumes as for the BOLD_STD_ calculations, using the MatLab toolbox DPARSFA^2^ (Chao-Gan and Yu-Feng, 2010). Thus, for the examined ALFF calculations, the examined frequency window was essentially the same as the bandpass filter used in the resting state data, i.e., 0.008–0.09 Hz.

#### Graph Analyses

For the network analyses, we created subject specific weighted graphs based on 264 nodes defined as spherical (4 mm radius) ROIs centered around functionally relevant coordinates (Power et al., 2011; Cole et al., 2013) (see Supplementary Table S1). Edges were defined as Pearson correlation values between each time series of each ROI–ROI pair, where negative correlations were nulled. For measures of hippocampal betweenness centrality, we added two nodes corresponding to the left and right hippocampus (see Seed Based Correlation Analysis) to the original (264 nodes) graph. A high degree of betweenness centrality imply that nodes have central positions, i.e., act as hubs in the given network. Global efficiency is a measure of how efficiently information is exchanged across a network. All graph theoretical measures (i.e., Betweenness Centrality for left and right hippocampus, and global efficiency) were calculated using the brain connectivity toolbox (Rubinov and Sporns, 2010) as implemented in GraphVar1.0 (Kruschwitz et al., 2015).

#### Independent Component Analyses

Independent component analysis (ICA) was performed using the conn toolbox (v16, Whitfield-Gabrieli and Nieto-Castanon, 2012). Comparable ICA’s across subjects were obtained through a dual regression procedure, where we first calculated 20 group level spatial independent components (default value), and subsequently used the time series pertaining to each component to obtain subject-specific spatial components. Longitudinal change in five different networks were analyzed with regard to baseline and change in aerobic capacity, respectively. The ICA components of interest were chosen in an attempt to conceptually replicate the analysis of resting state networks investigated in Voss et al. (2016). Specifically, these included the default mode network (DMN), right and left frontoparietal network (FPN), dorsal attention network (DAN), and Sensorimotor Network (S1M1) (see Supplementary Figure S1).

#### Network Based Statistics

We also conducted NBS analysis (Zalesky et al., 2010), on the graph consisting of the same 264 nodes as used for graph analysis (see Graph Analyses). NBS allows for identification of group-effects among ROI–ROI connections or subnetworks, while controlling for multiple comparisons. NBS is the conceptual equivalent to cluster statistics for voxel wise whole brain analyses. Analogous to cluster statistics, NBS yields stronger statistical sensitivity for distributed effects, which in turn is traded for spatial resolution which is limited to the size of the identified network (c.f. cluster), rather than individual ROI–ROI connections (c.f. voxels). NBS was used to identify ROI–ROI pairs or subnetworks that revealed group by time interactions, or where connectivity related to aerobic capacity (either at baseline, or in change-change regressions). For this, we employed a typically used network defining ROI–ROI (“primary”) connectivity threshold (Zalesky et al., 2010); *p* = 0.001 uncorrected. In order to detect weaker but spatially more distributed sub-graphs for 2nd level contrasts where the original ROI–ROI connectivity threshold did not show any effect, we also investigated fitness-network associations using ROI–ROI connectivity thresholds of *p* = 0.01 and *p* = 0.05 uncorrected (two sided). For NBS (i.e., “network component intensity”) threshold, we used the significance level of family-wise error correction (FWE) *p* = 0.05 (1000 permutations).

#### Whole Brain Pattern of Functional Connectivity

Additionally, we performed a principal component MVPA, or so called “connectome-MVPA,” to detect patterns of whole brain connectivity that correlated with aerobic capacity. The MVPA analysis complements the SCA and ICA since the investigated connectivity is not restricted to pre-selected seed regions or independent components, respectively. Instead, it provides a regionally unbiased mapping of brain areas with whole brain connectivity patterns that are predicted by aerobic capacity or longitudinal change thereof. In detail, the MVPA measure was obtained by dimension reduction of the whole brain connectivity matrix of each voxel. The connectivity matrix of each subject was reshaped into a row vector and subsequently concatenated over all participants into a matrix *N* × *V*, where *N* was the number of subjects and *V* is the number of voxels within the brain mask. The dimensionality of the *N* × *V* group correlation matrix was reduced by principal component analysis (PCA). This yielded an *N* × *C* matrix, where *C* is the number of maintained principal components. We maintained the first seven principal components that explained the most of the variance of the connectivity matrix (*C* = 7) [according to the rule of thumb to maintain an approximate 1:7 ratio between number of components and subjects, as proposed by the metric implementer Nieto-Castenon (2015)^3^]. In other words, the resulting seven component score volumes best represented the whole brain connectivity pattern for each subject. These volumes were included in an *F*-test at the 2nd level analysis. Thus, we tested for clusters that were predicted by aerobic capacity with regard to whole brain connectivity.

**FIGURE 1:**
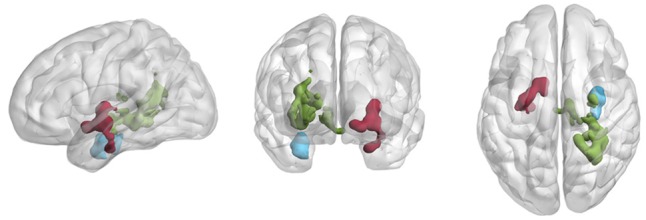
Relationship between aerobic capacity and fluctuations of BOLD signal (BOLD_STD)_ at baseline. For three regions in medial temporal areas, we observed a negative relationship between aerobic capacity and BOLD_STD_. Green: a cluster (973 voxels) extending parts of right posterior hippocampus and thalamus. Red: cluster (298 voxels) extending parts of L. Putamen, (l) thalamus and (2) amygdala. Blue: cluster (232 voxels) extending part of right anterior hippocampus and amygdala. Results are visualized using brain net viewer (Xia et al., 2013).

**FIGURE 2:**
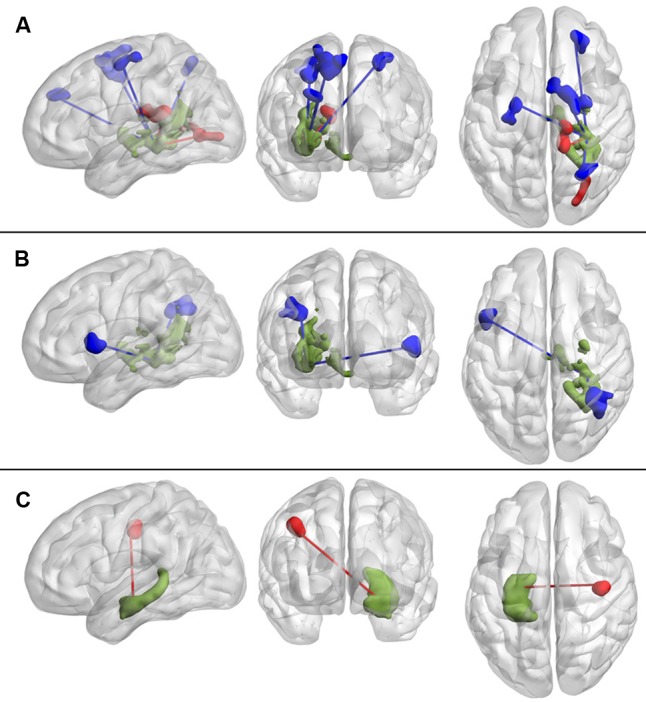
Seed based correlation analysis relating aerobic capacity to resting state functional connectivity. **(A)** At baseline, aerobic capacity (VO_2_-peak) was primarily positively (blue) correlated with right medial temporal lobe connectivity to frontal, parietal, and occipital areas. Negative correlations between aerobic capacity and connectivity of the same seed were observed to thalamus and occipital regions. **(B)** Longitudinal gains in aerobic capacity predicted increased functional connectivity between the right medial temporal lobe and frontal and parietal regions. **(C)** Change in aerobic capacity is negatively associated with connectivity between left hippocampus and contralateral precentral gyrus. Green areas indicate seed regions; blue indicate positive associations to (change in) aerobic capacity; red indicates negative associations.

**FIGURE 3:**
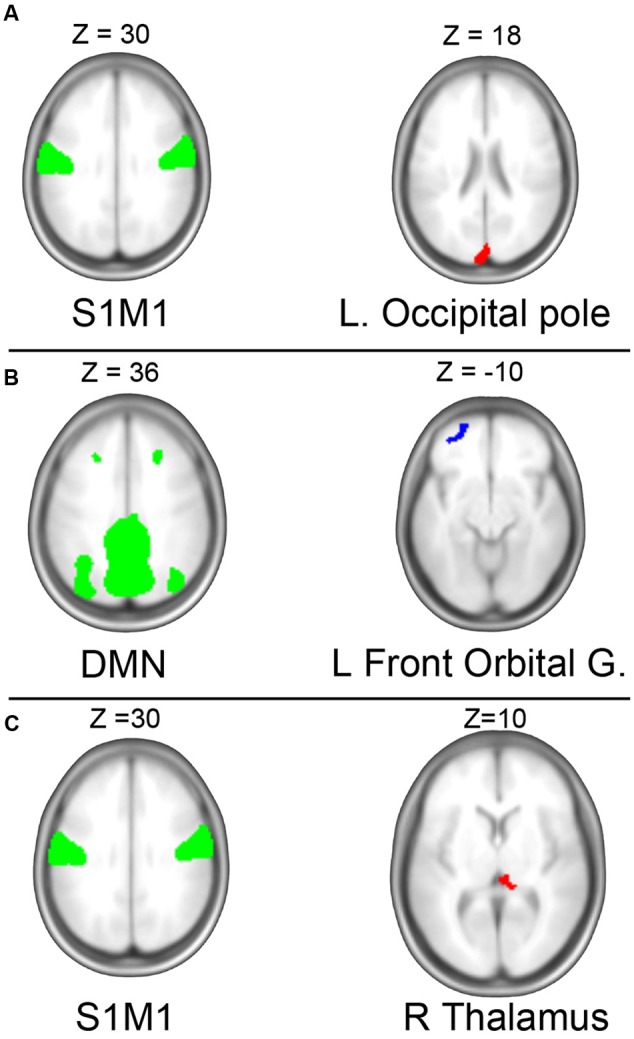
Independent component analysis (ICA). **(A)** ICA revealed a negative association between baseline measures of aerobic capacity and connectivity between the sensorimotor network (left) the occipital pole (right). **(B)** Longitudinal gain in aerobic capacity was associated with increased connectivity between default mode network (DMN) and left prefrontal cortex. **(C)** Change in VO_2_-peak was negatively related to change in connectivity of the somatosensory network (S1M1) to right thalamus. Green regions illustrate the independent components (IC) at baseline; red indicates areas experiencing decreased connectivity to the IC with higher (or gain in) VO_2_-peak; blue indicates areas with increased fitness predicted connectivity.

The reported activation maps (of contrasted regression parameter estimates) in group analyses were considered significant at a cluster-level significance of *p* < 0.05, false-discovery rate (FDR) corrected, and cluster defining voxel threshold of *p* < 0.001, two-sided. Due to unreliable estimations of smoothness of the MVPA rendered PCA maps, we used non-parametric permutation statistics (1000 permutations) as advocated by the implementer of the metric (Alfonso Nieto-Castanon, personal communication, September, 23, 2016). Connectome-MVPA analyses were thresholded using a cluster based significance threshold of *p* = 0.05 (FDR). Un-thresholded statistical maps for all reported results (**Figures 1–3**) are available online at http://neurovault.org/collections/2415/.

For all significant findings of group by time interactions, and associations between intrinsic brain activity either with regard to baseline aerobic capacity or change in aerobic capacity, we further examined the relationships to cognition. More specifically, we defined volumes of interest based on the brain regions that were significantly predicted by aerobic capacity. These volumes were subsequently used as inclusive masks (i.e., small volume correction) within which we investigated effects of either baseline score or longitudinal change in cognition, respectively.

## Results

### Intervention Induced Gains in Aerobic Capacity

Within the sub-sample used here we observed a significant group by time interaction, with the aerobic group displaying a significantly larger improvement in aerobic capacity compared to the active control group *F*(1,43) = 6.20, *p* = 0.02 (see **Table 1**). This was similar to what Jonasson et al. (2017) reported using the full sample.

### Intervention Induced Changes in Intrinsic Brain Activity

None of the analyses (described in the method fMRI Analysis) of intrinsic brain activity reveled significant group by time interactions, neither with nor without control for sex, age and baseline VO_2_-peak estimates.

Likewise, the NBS-analysis was non-significant using a range of network defining ROI–ROI thresholds (see Network Based Statistics). Finally, none of the graph theoretical measures (betweenness centrality of left and right hippocampus, or global efficiency) rendered significant group by time interactions, see **Table 1**.

Given non-significant significant group by time interactions with regard to intrinsic brain activity, we conducted additional exploratory analyses that in future may guide confirmatory studies. In these, we tested for relationships between longitudinal change in VO_2_-peak and intrinsic brain activity across all participants disregarding group belonging, similar to what has been done previously (Maass et al., 2015; Jonasson et al., 2017). Therefore, we first explored cross-sectional relationships between VO_2_-peak and brain activity at baseline. Any relationships observed at baseline data were subsequently used to inform regressions of longitudinal change in VO_2_-peak to changes in brain activity.

### Relationship between Aerobic Capacity with Resting State Measures at Baseline

#### BOLD-Signal Fluctuations

Variation of the BOLD signal time series (BOLD_STD_) was negatively correlated with aerobic capacity in three clusters located in midbrain regions at baseline (**Figure 1** and **Table 2**). The largest cluster (973 voxels) extended over right posterior hippocampus and thalamus. A second cluster (298 voxels) covered parts of left pallidum, and to a smaller extent also left thalamus and amygdala. The third cluster (232 voxels) contained right anterior hippocampus and right amygdala. Clusters were anatomically labeled using the WFU pick atlas. BOLD_STD_ were not positively correlated with aerobic capacity in any brain region.

**Table 2:** Significant relationships between BOLD_STD_ and aerobic capacity at baseline.

Contrast	Region (peak co-oridnates)	Cluster size (# voxels)	Cluster p-FDR
Neg pre VO_2_-peak	R. Hippocampus (32, -32, -8)	973	<0.000001
	L. Putamen (-22, -2, -6)	298	0.004
	R. Hippocampus (32, -9, 26)	232	0.002

Amplitude of Low Frequency Fluctuations (ALFF), resulted in virtually identical results as BOLD_STD_ (see Supplementary Figure S2), and thus ALFF results are not presented further.

Follow-up analyses that tested for the association between cognitive performance and BOLD_STD_ were restricted to the search volume of regions where aerobic capacity predicted BOLD_STD_ (displayed in **Figure 1**). Given that particularly hippocampal and mid-temporal brain regions displayed fitness related BOLD_STD_, we tested for EM. In addition, a meta-analysis (Colcombe and Kramer, 2003) found the largest association between improved fitness and cognitive performance to be related to EFs. Furthermore, in our previous study, improved fitness was primarily related to improved general cognitive performance (i.e., CS) (Jonasson et al., 2017). Therefore, we also tested the association between EF, CS and BOLD_STD_. Neither EM, EF nor CS were significantly related to BOLD_STD_. (All cognitive outcome measures are presented in detail in Jonasson et al., 2017).

#### Seed Correlation Analysis (SCA)

None of the *a priori* defined seeds displayed significant VO_2_-peak predicted connectivity at baseline. However, connectivity of the *post hoc* seed in the right medial temporal cortex (see BOLD-Signal Fluctuations), to frontal-, parietal-, and occipital regions displayed a positive correlation to aerobic capacity. We also detected a negative association between aerobic capacity and the connectivity between the same seed and right thalamus and right occipital cortex (**Figure 2A** and **Table 3**).

**Table 3:** Relationships between functional connectivity and aerobic capacity at baseline and change over time.

Contrast	Seed	Target(s) (peak coordinates)	Cluster size (# voxels)	Cluster p-FDR
Pre VO_2_-peak	R. Hippocampus^∗^	R. Superior Frontal Gyrus (14, 2, 58)	458	0.000005
		L. Precentral gyrus (-22, -6, 56)	132	0.014
		R. Occipital cortex (28, -62, 58)	116	0.017
		R. Frontal pole (22, 48, 32)	116	0.017
Neg pre VO_2_-peak	R. Hippocampus^∗^	R. Thalamus (WM), (12, -28, 18)	178	0.0047
		Occipital (WM) (22, -86, 0)	145	0.012
	ICA S1M1	L. Occipital Pole (2, -92, 18)	133	0.0019
dVO_2_-peak	R. Hippocampus^∗^	R. Angular Gyrus (36, -48, 30)	197	0.0013
		L. Inf Frontal Gyrus (-48, 14, 6)	105	0.022
	ICA DMN	L. Front. Orbital (-32, 30, -8)	109	0.019
Neg dVO_2_-peak	L. Hippocampus	R. Precentral Gyrus (-36, -12, 36)	139	0.0097
	ICA S1M1	R. Thalamus (-6, -28, 12)	114	0.0043

*Target regions are labeled based on the locations of the largest number of voxels within significant cluster, as identified and labeled within the Conn-toolbox. ^∗^Seed region defined *post hoc* from VO_*2*_-peak predicted BOLD_*STD*_ effects at baseline.*

#### Independent Component Analysis (ICA)

At baseline, among the five investigated ICA-derived networks we observed a negative relationship between VO_2_-peak and the connectivity of a sensorimotor network and a brain region extending over calcarine sulcus and precuneus (see **Figure 3A**). No associations between VO_2_-peak and connectivity of the other four networks were seen.

#### Graph Measures, NBS, Connectome-MVPA and ASL

Several measures of intrinsic brain activity turned out not to be significantly related to aerobic capacity. For graph theoretical measures, neither global efficiency [*t*(42) = -0.06, *p* = 0.95] nor left or right hippocampal centrality [L: *t*(42) = -0.94, *p* = 0.34, R: *t*(42) = -1.23, *p* = 0.22] reached significance.

In the NBS analysis, no ROI–ROI connectivities or subnetworks were correlated with aerobic capacity, neither corrected nor uncorrected. Furthermore, the connectome-MVPA did not reveal any significant clusters predicted by baseline VO_2_-peak.

Finally, analysis of ASL perfusion data did not reveal any associations between aerobic capacity and CBF from neither whole brain nor small volume voxel based analysis.

### Relationship between Longitudinal Change in Aerobic Capacity and Change in Intrinsic Brain Activity

#### Seed Correlation Analysis

Longitudinal gain in aerobic capacity was positively associated with changes in functional connectivity between the BOLD_STD_-derived seed region in right medial temporal lobe, and right angular gyrus and left inferior frontal gyrus (**Figure 2B** and **Table 3**). However, change in aerobic capacity was negatively associated with change in connectivity between a priori defined seed regions in the left hippocampus and the right precentral gyrus (**Figure 2C** and **Table 3**). None of the other a priori defined seeds related to change in VO_2_-peak.

Behavioral follow-up analyses did not reveal any significant relationships between changes in the fitness related SCA results and EM, EF, or CS.

#### Independent Component Analysis

Independent component analysis identified two significant associations between changes in VO_2_-peak and connectivity. Firstly, there was a positive relationship between gains in VO_2_-peak and connectivity between DMN and left frontal pole (see **Figure 3B** and **Table 3**). Secondly, a negative relationship between change in VO_2_-peak and the connectivity between the S1M1-network and right hypothalamus was observed (**Figure 3C**). There was no significant relationship to changes in cognition (EM, EF, and CS).

#### Network Based Statistics

**FIGURE 4:**
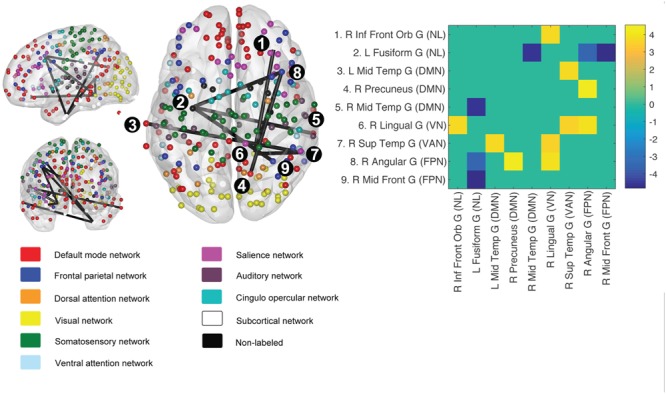
Network based statistics (NBS) derived graph component predicted by change in VO_2_-peak. Using change in VO2-peak to predict longitudinal change in the brain graph (264 nodes), we retrieved a subgraph spanning both task-negative and task-positive networks. Edge definition was *p* = 0.001 unc, and network component significance threshold FWE corrected *p* < 0.05. The adjacency matrix (top right) depicts participating nodes, and only significant *t*-values (*t* > 3.54) are shown. For MNI-coordinates of participating nodes, see Supplementary Table S1.

The NBS revealed a subnetwork constituted by connected nodes in the DMN, the frontal parietal-, visual- and ventral attention network (**Figure 4**) in which gains in fitness was related to changes in intra-network connectivity. Since the subgraph detected by NBS does not allow for inferences on individual edges (i.e., ROI–ROI connections), we also correlated change in VO_2_-peak to connectivity between all individual ROI–ROI pairs (i.e., 34584 individual connections). In this analysis, no connections were significant (Bonferroni correcting for the edges in the full adjacency matrix).

#### Graph Measures, BOLD_STD_, Connectome-MVPA and ASL

For the graph analysis, neither global efficiency [*F*(1,42) = 0.70, *p* = 0.39], nor betweenness centrality of hippocampus [left: *F*(1,43) = 1.35, *p* = 0.25; right: *F*(1,43) = 0.45, *p* = 0.50] was significantly related to change in fitness.

None of the other measures of intrinsic brain activity (MVPA, BOLD_STD_,) or ASL yielded significant change-change relationships to fitness.

## Discussion

In the current study, we aimed to characterize the effects of exercise induced improvements in aerobic capacity on intrinsic brain activity. To accomplish this, we have employed a wide range of resting-state fMRI metrics as well as measures of CBF using ASL.

### No Longitudinal Group Differences in Brain Activity

Based on previous literature, we predicted significant group by time interactions with regard to hippocampal connectivity, DMN integrity and vascular response as revealed by BOLD_STD_ and CBF. In the present study, none of these measures displayed group differences in longitudinal changes.

A possible explanation may be that both groups improved in aerobic capacity. Since we stipulate that gain in VO_2_-peak would be the active constituent mediating improvements in cognition and modulations of intrinsic brain activity (in line with the fitness hypothesis of cognition, (North et al., 1990; Kramer et al., 1999)], we reasoned that interindividual change in aerobic capacity across both groups could be a sensitive predictor of brain changes (similar to approaches used in previous studies, e.g., Maass et al., 2015). Thus, we performed *post hoc* analyses investigating inter individual change–change relations. The lack of significant effects is discussed in further detail below.

### *Post hoc* Analyses of Fitness-Brain Relationships

#### Aerobic Capacity and the Medial Temporal Lobe

In the *post hoc* analyses we observed several associations between aerobic fitness and intrinsic brain activity, both at baseline and over time. Longitudinal change in aerobic capacity predicted change in hippocampal connectivity. Whereas left hippocampus displayed a decreased connectivity to precentral gyrus with increasing fitness, the right hippocampus was associated with increased connectivity to frontal and parietal areas. The right hippocampus, similar to baseline, was associated with increased frontal and parietal connectivity. Previously, Voss et al. (2013) reported enhanced connectivity between parahippocampus and bilateral temporal cortex, as well as to occipital and parietal areas following a 12 months aerobic (walking) intervention. Similar to the current findings, they observed reduced connectivity of left hippocampus, although to the right prefrontal cortex rather than to the right precentral gyrus as seen in here. Interestingly, the authors showed that increased connectivity between bilateral parahippocampal gyrus and middle temporal gyri were positively predicted by increases in neurotrophic growth factors, although no direct test of the behavioral significance of any of the connectivity changes was presented.

We detected relationships between BOLD_STD_ and aerobic capacity at baseline, whereas gains in aerobic capacity did not relate to changes in BOLD_STD_ over time. Contrary to what previously has been reported (Burzynska et al., 2015; Gauthier et al., 2015), the relationships between aerobic fitness and BOLD_STD_ were negative and primarily observed in mid-temporal rather than cortical areas. Burzynska et al. (2015) reported cross-sectional associations between BOLD_STD_ and physical activity, suggesting that BOLD_STD_ could reflect a long-term cardiovascular trait, which coheres with the absence of any intervention related changes in current study. Notably, they did not observe relationship between aerobic capacity and BOLD_STD_, but only a positive relationship between BOLD_STD_ and physical activity. Our findings suggest that high aerobic capacity is associated with a more stable BOLD-signal within the temporal medial lobe. Speculative, the high fit subjects would experience relatively lower levels of physical demand, which could reconcile current findings with those reported by Burzynska et al. (2015). However, no on-line monitoring of degree of physical work during scanning was performed. Thus, further studies are required to establish the relationship between gains in aerobic capacity and BOLD_STD_.

Longitudinal changes in intrinsic brain activity that were predicted by change in aerobic fitness did not relate to changes in any of the investigated cognitive domains (EM, EF, and CS). A plausible reason for this is the absent relationships between the cognitive variables and VO_2_-peak, both at baseline and in change-change scores (Supplementary Table S2). Unfortunately, this limits the conclusions of the functional meaning of the observed fitness related brain changes. Thus, any functional interpretations of these brain changes are unknown and would have to rely on reverse inference, for which we lack unique cognition-connectivity associations.

Taken together, the findings above support the notion that aerobic fitness primarily affects mid temporal brain regions. In addition to the exercise intervention studies investigating changes in intrinsic brain activity (Voss et al., 2013; Burzynska et al., 2015; Maass et al., 2015; Bär et al., 2016; Tozzi et al., 2016), corroborating evidence of hippocampal involvement is also provided by studies investigating structural changes (e.g., Erickson et al., 2011, for a review, see Erickson et al., 2014; Jonasson et al., 2017). The fact that hippocampus display a high capacity for plastic change in relation to environmental factors (for a review, see Leuner and Gould, 2010), and also undergoes age related resting state connectivity changes (Salami et al., 2016), provides further motivation to specifically probe the intrinsic brain activity in the mid-temporal lobe. By standardizing research protocols and outcome measures, the mechanisms linking hippocampal brain activity, brain structure, vascularity, molecular growth factors and behavior will hopefully be revealed (Duzel et al., 2016; Stillman et al., 2016).

#### Network Changes

Network based statistics analysis revealed a graph sub-component that was predicted by the longitudinal change in VO_2_-peak. Whereas previous cross-sectional reports (Voss et al., 2016) showed a higher within network integrity (particularly in DMN) with higher fitness, our longitudinal findings suggest that increased fitness also modulate between network connectivity. Whether the explanations for the discrepant finding are found in differences in methodological approaches (e.g., graph defining set of nodes) or lack of statistical power remains to be determined.

Resting state networks derived by ICA revealed relationships between fitness and connectivity of both the S1M1 and the DMN. Decreased connectivity between M1S1 and right thalamus was associated with increased aerobic capacity. M1S1 receives input from parts of thalamus, and the decreased connectivity with gains in fitness seems counterintuitive at first. Speculatively, this could reflect enhanced neuronal efficiency, although such a hypothesis should be investigated using methods that complement the correlational fMRI approaches used here. The connectivity between the same S1M1 network and occipital cortex were negatively related to fitness at baseline. In a small exercise intervention among obese children, Krafft et al. (2014) observed that the ICA derived motor network underwent decreased connectivity to visual areas in precuneus in the exercise group, resembling current baseline finding. Although our fitness predicted S1M1 connectivity targeted different regions at baseline compared to over time, it seems that functional segregation of S1M1 reflects better fitness. However, in an aged cohort more similar to ours, Voss et al. (2010) did not see group by time changes in motor connectivity, why this finding would need to be investigated further.

Interestingly, we observed enhanced connectivity between the DMN component and the left orbitofrontal cortex that correlated with gains in fitness. Even though the orbital cluster is localized more laterally than the typical ventromedial frontal DMN hub, the observation bears similarities to previous literature of fitness related connectivity of the posterior anterior midline core of the DMN (Voss et al., 2010, 2016). Both these studies interpret the fitness-related DMN integrity as a rejuvenated connectivity pattern, typical for younger subjects. A similar interpretation could be given here, but the lack of associations between cognition and these fitness-related connectivity patters prevent any firm conclusion of their behavioral significance.

### Limited Effects

The *a priori* hypothesized group by time interactions, as well as the majority of the exploratory tests that aimed to relate longitudinal change in VO_2_-peak to change in intrinsic brain activity, turned out to be non-significant. Despite drawing on previous literature that have investigated intrinsic capacity in relation to aerobic fitness among elderly, we were largely unable to conceptually replicate these. The replication failures could likely be attributed both to factors pertaining to cognitive brain imaging in general (see below), as well as to factors specific to this study context (e.g., differences in study design, interventions, cohorts and analyses strategies). Another potentially important issue is the fact that the active control group may have exercised more vigorously than in many previous interventions, considering that aerobic capacity increased substantially also for this group. Recent research have shown that resistance and coordination training, part of our active control training regimen, may influence the BOLD-signal during task performance (Voelcker-Rehage et al., 2011; see Voelcker-Rehage and Niemann, 2013, for a review), as well as show similar effects on hippocampus volume as aerobic exercise (Niemann et al., 2014). By comparing two training regimens which both have positive effects on brain and behavior, actual intervention effects may thus have been masked.

The effects of gains in fitness on the exploratory resting-state measures (e.g., global efficiency, connectome-MVPA, BOLD_STD_) of the resting-state brain activity were likely too subtle to be detected in current study. The BOLD signal acquired during resting state fMRI is inherently noisy, and estimations based on high quality fMRI data showed that the variance associated with neuronal activity only constituted around 4% of the total variance of the BOLD-signal time series (Marcus et al., 2013). However, despite the large contribution of non-neuronal noise to the resting state BOLD signal, test–retest reliability of group average of cardinal resting state networks has been reported to be fairly reliable over time (Zuo et al., 2010; Wisner et al., 2013), although the choice of both resting state measures and preprocessing pipeline influence the degree of test–retest reliability (Yan et al., 2013). Not surprisingly, between-group comparisons of brain activity are also typically much smaller than single group averages (for an informative discussion on effect sizes in fMRI, see Poldrack et al., 2017).

### Limitations and Future Directions

Current study has several limitations. First, we were unable to selectively induce improvements of VO_2_-peak in the aerobic group, although the aerobic group improved more than the control group. This likely decreased any exercise induced group differences of the outcome measures. Although challenging, experimental designs that more efficiently prevent cardiovascular training in the active control group could potentially reveal stronger experimentally induced brain changes.

Furthermore, current study only measured two time points, i.e., before and after the 6-months intervention. Future studies would benefit from sampling data at multiple (>2) time points, for higher temporal resolution of the developmental trajectories. One motive for this is the expansion-partial normalization hypothesis of neuroplasticity (Brehmer et al., 2014), which proposes that longitudinal brain changes commonly follow inverted u-shaped temporal profiles, which could only be detected if data were acquired at multiple time points.

Resting state studies of physical interventions are still sparse. A greater understanding of the mechanisms mediating the neurocognitive effects of aerobic exercise are valuable for optimizing intervention programs to target the relevant neurophysiological processes and cognitive domains with higher precision. To enhance the reliability and replicability of imaging findings, several factors have recently been proposed (Ioannidis et al., 2014; Poldrack et al., 2017). Complete reporting of results (i.e., including null findings), as well as clear declarations of which analyses are *ad hoc* or exploratory and which are hypothesis driven, are both critical when aggregating research findings in meta-analyses, or for informing the design of future studies. Above all, the authors emphasized the importance of attaining proper statistical power, e.g., by enhancing cohort sizes for instance through laudable data sharing initiatives. Moreover, increased statistical power is also obtained by larger effect sizes. Stronger experimental effects could likely be achieved by longer and more intensive interventions. Potentially, longer intervention studies would also enable the observed brain changes to translate into improvements in behavior.

## Conclusion

We have characterized changes in intrinsic brain activity following a 6-months physical exercise intervention. None of our *a priori* hypothesis of group by time interactions regarding intrinsic brain activity were confirmed. However, when investigating the linear relationships between longitudinal gain in aerobic capacity and changes in functional connectivity we observed exercise modulated hippocampal connectivity. Likewise, gain in aerobic capacity was associated with increased connectivity between the DMN and prefrontal cortex, but negatively related to sensorimotor-thalamic connectivity. However, these changes did not relate to changes in cognition, possibly due to the length of the intervention, or insensitive behavioral measures. The majority of the exploratory analyses did not reveal significant associations between fitness and intrinsic brain activity, although we observed that mid-temporal BOLD_STD_ was negatively associated with fitness at baseline. The current study provides resting-state fMRI evidence that exercise preferentially modulate mid-temporal brain regions and hippocampus. The functional significance of these brain changes should be investigated further, preferably in high powered studies using data acquired at multiple time points.

## Ethics Statement

This study was carried out in accordance with the recommendations of the regional ethical committee in Umeå, Sweden with written informed consent from all subjects. All subjects gave written informed consent in accordance with the Declaration of Helsinki. The protocol was approved by the regional ethical committee in Umeå, Sweden.

## Author Contributions

LJ, LN, KR, and CB designed the study. PF performed the statistical analyses. All authors contributed in revising the work and approved the final version of the manuscript.

## Conflict of Interest Statement

The authors declare that the research was conducted in the absence of any commercial or financial relationships that could be construed as a potential conflict of interest.

## Footnotes

1 http://www.fmrib.ox.ac.uk/fsl

2 http://restfmri.net

3 http://www.nitrc.org/forum/message.php?msg_id=14332
