# Supplementary material for: Does Aerobic Exercise Influence Intrinsic Brain Activity? An Aerobic Exercise Intervention among Healthy Old Adults
Source: Front Aging Neurosci. 2017 Aug 11;9:267. doi: 10.3389/fnagi.2017.00267 (PMC5554511; doi:10.3389/fnagi.2017.00267)
Supplement: Supplementary file 1 [file Table_1.DOCX]

**Supplemental Table S1**. *Brain regions used as nodes in graph analysis*. Seeds are adapted from Power et al (2011).

| ROI# | RSN | Brain Region | x | y | z | ID of hubs in fig 4 |
| --- | --- | --- | --- | --- | --- | --- |
| 1 | NL | Occipital_Inf_L (aal) | -25 | -98 | -12 |  |
| 2 | NL | Lingual_R (aal) | 27 | -97 | -13 |  |
| 3 | NL | Frontal_Sup_Orb_R (aal) | 24 | 32 | -18 |  |
| 4 | NL | Temporal_Inf_L (aal) | -56 | -45 | -24 |  |
| 5 | NL | Rectus_R (aal) | 8 | 41 | -24 |  |
| 6 | NL | undefined | -21 | -22 | -20 |  |
| 7 | NL | ParaHippocampal_R (aal) | 17 | -28 | -17 |  |
| 8 | NL | Fusiform_L (aal) | -37 | -29 | -26 |  |
| 9 | NL | Temporal_Inf_R (aal) | 65 | -24 | -19 |  |
| 10 | NL | Temporal_Inf_R (aal) | 52 | -34 | -27 |  |
| 11 | NL | Temporal_Inf_R (aal) | 55 | -31 | -17 |  |
| 12 | NL | Frontal_Inf_Orb_R (aal) | 34 | 38 | -12 | 1 |
| 13 | somato-motor | Precuneus_L (aal) | -7 | -52 | 61 |  |
| 14 | somato-motor | undefined | -14 | -18 | 40 |  |
| 15 | somato-motor | Supp_Motor_Area_L (aal) | 0 | -15 | 47 |  |
| 16 | somato-motor | Supp_Motor_Area_R (aal) | 10 | -2 | 45 |  |
| 17 | somato-motor | Paracentral_Lobule_L (aal) | -7 | -21 | 65 |  |
| 18 | somato-motor | Paracentral_Lobule_L (aal) | -7 | -33 | 72 |  |
| 19 | somato-motor | Postcentral_R (aal) | 13 | -33 | 75 |  |
| 20 | somato-motor | Parietal_Inf_L (aal) | -54 | -23 | 43 |  |
| 21 | somato-motor | Precentral_R (aal) | 29 | -17 | 71 |  |
| 22 | somato-motor | Precuneus_R (aal) | 10 | -46 | 73 |  |
| 23 | somato-motor | Postcentral_L (aal) | -23 | -30 | 72 |  |
| 24 | somato-motor | Precentral_L (aal) | -40 | -19 | 54 |  |
| 25 | somato-motor | Postcentral_R (aal) | 29 | -39 | 59 |  |
| 26 | somato-motor | Postcentral_R (aal) | 50 | -20 | 42 |  |
| 27 | somato-motor | Postcentral_L (aal) | -38 | -27 | 69 |  |
| 28 | somato-motor | undefined | 20 | -29 | 60 |  |
| 29 | somato-motor | Precentral_R (aal) | 44 | -8 | 57 |  |
| 30 | somato-motor | Postcentral_L (aal) | -29 | -43 | 61 |  |
| 31 | somato-motor | Supp_Motor_Area_R (aal) | 10 | -17 | 74 |  |
| 32 | somato-motor | Postcentral_R (aal) | 22 | -42 | 69 |  |
| 33 | somato-motor | Postcentral_L (aal) | -45 | -32 | 47 |  |
| 34 | somato-motor | Postcentral_L (aal) | -21 | -31 | 61 |  |
| 35 | somato-motor | Paracentral_Lobule_L (aal) | -13 | -17 | 75 |  |
| 36 | somato-motor | Precentral_R (aal) | 42 | -20 | 55 |  |
| 37 | somato-motor | undefined | -38 | -15 | 69 |  |
| 38 | somato-motor | Parietal_Sup_L (aal) | -16 | -46 | 73 |  |
| 39 | somato-motor | Paracentral_Lobule_R (aal) | 2 | -28 | 60 |  |
| 40 | somato-motor | Supp_Motor_Area_R (aal) | 3 | -17 | 58 |  |
| 41 | somato-motor | Precentral_R (aal) | 38 | -17 | 45 |  |
| 42 | somato-motor | Postcentral_L (aal) | -49 | -11 | 35 |  |
| 43 | somato-motor | Insula_R (aal) | 36 | -9 | 14 |  |
| 44 | somato-motor | Postcentral_R (aal) | 51 | -6 | 32 |  |
| 45 | somato-motor | Postcentral_L (aal) | -53 | -10 | 24 |  |
| 46 | somato-motor | Postcentral_R (aal) | 66 | -8 | 25 |  |
| 47 | CON | Supp_Motor_Area_L (aal) | -3 | 2 | 53 |  |
| 48 | CON | SupraMarginal_R (aal) | 54 | -28 | 34 |  |
| 49 | CON | Frontal_Sup_R (aal) | 19 | -8 | 64 |  |
| 50 | CON | Frontal_Sup_L (aal) | -16 | -5 | 71 |  |
| 51 | CON | Cingulum_Mid_L (aal) | -10 | -2 | 42 |  |
| 52 | CON | undefined | 37 | 1 | -4 |  |
| 53 | CON | Supp_Motor_Area_R (aal) | 13 | -1 | 70 |  |
| 54 | CON | Supp_Motor_Area_R (aal) | 7 | 8 | 51 |  |
| 55 | CON | Rolandic_Oper_L (aal) | -45 | 0 | 9 |  |
| 56 | CON | Insula_R (aal) | 49 | 8 | -1 |  |
| 57 | CON | undefined | -34 | 3 | 4 |  |
| 58 | CON | Temporal_Pole_Sup_L (aal) | -51 | 8 | -2 |  |
| 59 | CON | Cingulum_Mid_L (aal) | -5 | 18 | 34 |  |
| 60 | CON | undefined | 36 | 10 | 1 |  |
| 61 | auditory | undefined | 32 | -26 | 13 |  |
| 62 | auditory | Temporal_Sup_R (aal) | 65 | -33 | 20 |  |
| 63 | auditory | Temporal_Sup_R (aal) | 58 | -16 | 7 |  |
| 64 | auditory | Rolandic_Oper_L (aal) | -38 | -33 | 17 |  |
| 65 | auditory | Temporal_Sup_L (aal) | -60 | -25 | 14 |  |
| 66 | auditory | Temporal_Sup_L (aal) | -49 | -26 | 5 |  |
| 67 | auditory | Rolandic_Oper_R (aal) | 43 | -23 | 20 |  |
| 68 | auditory | SupraMarginal_L (aal) | -50 | -34 | 26 |  |
| 69 | auditory | SupraMarginal_L (aal) | -53 | -22 | 23 |  |
| 70 | auditory | Rolandic_Oper_L (aal) | -55 | -9 | 12 |  |
| 71 | auditory | Rolandic_Oper_R (aal) | 56 | -5 | 13 |  |
| 72 | auditory | Postcentral_R (aal) | 59 | -17 | 29 |  |
| 73 | auditory | undefined | -30 | -27 | 12 |  |
| 74 | DMN | Occipital_Mid_L (aal) | -41 | -75 | 26 |  |
| 75 | DMN | Frontal_Med_Orb_R (aal) | 6 | 67 | -4 |  |
| 76 | DMN | Rectus_R (aal) | 8 | 48 | -15 |  |
| 77 | DMN | Precuneus_L (aal) | -13 | -40 | 1 |  |
| 78 | DMN | Frontal_Sup_Orb_L (aal) | -18 | 63 | -9 |  |
| 79 | DMN | Temporal_Mid_L (aal) | -46 | -61 | 21 |  |
| 80 | DMN | Occipital_Mid_R (aal) | 43 | -72 | 28 |  |
| 81 | DMN | Temporal_Pole_Mid_L (aal) | -44 | 12 | -34 |  |
| 82 | DMN | Temporal_Pole_Mid_R (aal) | 46 | 16 | -30 |  |
| 83 | DMN | Temporal_Mid_L (aal) | -68 | -23 | -16 | 3 |
| 84 | NL | Temporal_Mid_L (aal) | -58 | -26 | -15 |  |
| 85 | NL | Insula_R (aal) | 27 | 16 | -17 |  |
| 86 | DMN | Angular_L (aal) | -44 | -65 | 35 |  |
| 87 | DMN | Angular_L (aal) | -39 | -75 | 44 |  |
| 88 | DMN | Precuneus_L (aal) | -7 | -55 | 27 |  |
| 89 | DMN | Precuneus_R (aal) | 6 | -59 | 35 |  |
| 90 | DMN | Precuneus_L (aal) | -11 | -56 | 16 |  |
| 91 | DMN | Precuneus_L (aal) | -3 | -49 | 13 |  |
| 92 | DMN | Cingulum_Mid_R (aal) | 8 | -48 | 31 |  |
| 93 | DMN | Precuneus_R (aal) | 15 | -63 | 26 | 4 |
| 94 | DMN | Cingulum_Mid_L (aal) | -2 | -37 | 44 |  |
| 95 | DMN | Precuneus_R (aal) | 11 | -54 | 17 |  |
| 96 | DMN | Angular_R (aal) | 52 | -59 | 36 |  |
| 97 | DMN | Frontal_Sup_R (aal) | 23 | 33 | 48 |  |
| 98 | DMN | Frontal_Sup_Medial_L (aal) | -10 | 39 | 52 |  |
| 99 | DMN | Frontal_Sup_L (aal) | -16 | 29 | 53 |  |
| 100 | DMN | Frontal_Mid_L (aal) | -35 | 20 | 51 |  |
| 101 | DMN | Frontal_Sup_R (aal) | 22 | 39 | 39 |  |
| 102 | DMN | Frontal_Sup_R (aal) | 13 | 55 | 38 |  |
| 103 | DMN | Frontal_Sup_L (aal) | -10 | 55 | 39 |  |
| 104 | DMN | Frontal_Sup_L (aal) | -20 | 45 | 39 |  |
| 105 | DMN | Frontal_Sup_Medial_R (aal) | 6 | 54 | 16 |  |
| 106 | DMN | Frontal_Sup_Medial_R (aal) | 6 | 64 | 22 |  |
| 107 | DMN | Cingulum_Ant_L (aal) | -7 | 51 | -1 |  |
| 108 | DMN | Frontal_Sup_Medial_R (aal) | 9 | 54 | 3 |  |
| 109 | DMN | Frontal_Med_Orb_L (aal) | -3 | 44 | -9 |  |
| 110 | DMN | Frontal_Med_Orb_R (aal) | 8 | 42 | -5 |  |
| 111 | DMN | Cingulum_Ant_L (aal) | -11 | 45 | 8 |  |
| 112 | DMN | Frontal_Sup_Medial_L (aal) | -2 | 38 | 36 |  |
| 113 | DMN | Cingulum_Ant_L (aal) | -3 | 42 | 16 |  |
| 114 | DMN | Frontal_Sup_L (aal) | -20 | 64 | 19 |  |
| 115 | DMN | Frontal_Sup_Medial_L (aal) | -8 | 48 | 23 |  |
| 116 | DMN | Temporal_Mid_R (aal) | 65 | -12 | -19 |  |
| 117 | DMN | Temporal_Mid_L (aal) | -56 | -13 | -10 |  |
| 118 | DMN | Temporal_Mid_L (aal) | -58 | -30 | -4 |  |
| 119 | DMN | Temporal_Mid_R (aal) | 65 | -31 | -9 | 5 |
| 120 | DMN | Temporal_Mid_L (aal) | -68 | -41 | -5 |  |
| 121 | DMN | Frontal_Sup_Medial_R (aal) | 13 | 30 | 59 |  |
| 122 | DMN | Cingulum_Ant_R (aal) | 12 | 36 | 20 |  |
| 123 | DMN | Temporal_Mid_R (aal) | 52 | -2 | -16 |  |
| 124 | DMN | ParaHippocampal_L (aal) | -26 | -40 | -8 |  |
| 125 | DMN | Fusiform_R (aal) | 27 | -37 | -13 |  |
| 126 | DMN | Fusiform_L (aal) | -34 | -38 | -16 |  |
| 127 | DMN | Cerebelum_Crus1_R (aal) | 28 | -77 | -32 |  |
| 128 | DMN | Temporal_Pole_Mid_R (aal) | 52 | 7 | -30 |  |
| 129 | DMN | Temporal_Mid_L (aal) | -53 | 3 | -27 |  |
| 130 | DMN | Angular_R (aal) | 47 | -50 | 29 |  |
| 131 | DMN | Temporal_Mid_L (aal) | -49 | -42 | 1 |  |
| 132 | NL | Frontal_Inf_Orb_L (aal) | -31 | 19 | -19 |  |
| 133 | NL | Cingulum_Post_L (aal) | -2 | -35 | 31 |  |
| 134 | NL | Precuneus_L (aal) | -7 | -71 | 42 |  |
| 135 | NL | Precuneus_R (aal) | 11 | -66 | 42 |  |
| 136 | NL | Precuneus_R (aal) | 4 | -48 | 51 |  |
| 137 | DMN | Frontal_Inf_Orb_L (aal) | -46 | 31 | -13 |  |
| 138 | VAN | Supp_Motor_Area_L (aal) | -10 | 11 | 67 |  |
| 139 | DMN | Frontal_Inf_Orb_R (aal) | 49 | 35 | -12 |  |
| 140 | NL | Lingual_R (aal) | 8 | -91 | -7 |  |
| 141 | NL | Lingual_R (aal) | 17 | -91 | -14 |  |
| 142 | NL | Lingual_L (aal) | -12 | -95 | -13 |  |
| 143 | visual | Lingual_R (aal) | 18 | -47 | -10 | 6 |
| 144 | visual | Occipital_Mid_R (aal) | 40 | -72 | 14 |  |
| 145 | visual | Calcarine_R (aal) | 8 | -72 | 11 |  |
| 146 | visual | Calcarine_L (aal) | -8 | -81 | 7 |  |
| 147 | visual | Occipital_Mid_L (aal) | -28 | -79 | 19 |  |
| 148 | visual | Lingual_R (aal) | 20 | -66 | 2 |  |
| 149 | visual | Occipital_Mid_L (aal) | -24 | -91 | 19 |  |
| 150 | visual | Fusiform_R (aal) | 27 | -59 | -9 |  |
| 151 | visual | Lingual_L (aal) | -15 | -72 | -8 |  |
| 152 | visual | Calcarine_L (aal) | -18 | -68 | 5 |  |
| 153 | visual | Occipital_Inf_R (aal) | 43 | -78 | -12 |  |
| 154 | visual | Occipital_Inf_L (aal) | -47 | -76 | -10 |  |
| 155 | visual | Occipital_Sup_L (aal) | -14 | -91 | 31 |  |
| 156 | visual | Cuneus_R (aal) | 15 | -87 | 37 |  |
| 157 | visual | Occipital_Mid_R (aal) | 29 | -77 | 25 |  |
| 158 | visual | Lingual_R (aal) | 20 | -86 | -2 |  |
| 159 | visual | Cuneus_R (aal) | 15 | -77 | 31 |  |
| 160 | visual | Lingual_L (aal) | -16 | -52 | -1 |  |
| 161 | visual | Temporal_Inf_R (aal) | 42 | -66 | -8 |  |
| 162 | visual | Occipital_Sup_R (aal) | 24 | -87 | 24 |  |
| 163 | visual | Cuneus_R (aal) | 6 | -72 | 24 |  |
| 164 | visual | Occipital_Mid_L (aal) | -42 | -74 | 0 |  |
| 165 | visual | Fusiform_R (aal) | 26 | -79 | -16 |  |
| 166 | visual | Cuneus_L (aal) | -16 | -77 | 34 |  |
| 167 | visual | Cuneus_L (aal) | -3 | -81 | 21 |  |
| 168 | visual | Occipital_Mid_L (aal) | -40 | -88 | -6 |  |
| 169 | visual | Occipital_Mid_R (aal) | 37 | -84 | 13 |  |
| 170 | visual | Calcarine_R (aal) | 6 | -81 | 6 |  |
| 171 | visual | Occipital_Mid_L (aal) | -26 | -90 | 3 |  |
| 172 | visual | Fusiform_L (aal) | -33 | -79 | -13 |  |
| 173 | visual | Occipital_Mid_R (aal) | 37 | -81 | 1 |  |
| 174 | FPN | Precentral_L (aal) | -44 | 2 | 46 |  |
| 175 | FPN | Frontal_Inf_Tri_R (aal) | 48 | 25 | 27 |  |
| 176 | FPN | Frontal_Inf_Tri_L (aal) | -47 | 11 | 23 |  |
| 177 | FPN | Parietal_Inf_L (aal) | -53 | -49 | 43 |  |
| 178 | FPN | Frontal_Mid_L (aal) | -23 | 11 | 64 |  |
| 179 | FPN | Temporal_Inf_R (aal) | 58 | -53 | -14 |  |
| 180 | FPN | Frontal_Sup_Orb_R (aal) | 24 | 45 | -15 |  |
| 181 | NL | Frontal_Mid_Orb_R (aal) | 34 | 54 | -13 |  |
| 182 | NL | Frontal_Mid_Orb_L (aal) | -21 | 41 | -20 |  |
| 183 | NL | Cerebelum_Crus1_L (aal) | -18 | -76 | -24 |  |
| 184 | NL | Cerebelum_Crus2_R (aal) | 17 | -80 | -34 |  |
| 185 | NL | Cerebelum_Crus1_R (aal) | 35 | -67 | -34 |  |
| 186 | FPN | Precentral_R (aal) | 47 | 10 | 33 |  |
| 187 | FPN | Precentral_L (aal) | -41 | 6 | 33 |  |
| 188 | FPN | Frontal_Mid_L (aal) | -42 | 38 | 21 |  |
| 189 | FPN | Frontal_Mid_R (aal) | 38 | 43 | 15 |  |
| 190 | FPN | Parietal_Inf_R (aal) | 49 | -42 | 45 |  |
| 191 | FPN | Parietal_Inf_L (aal) | -28 | -58 | 48 |  |
| 192 | FPN | Parietal_Inf_R (aal) | 44 | -53 | 47 |  |
| 193 | FPN | Frontal_Mid_R (aal) | 32 | 14 | 56 |  |
| 194 | FPN | Angular_R (aal) | 37 | -65 | 40 |  |
| 195 | FPN | Parietal_Inf_L (aal) | -42 | -55 | 45 |  |
| 196 | FPN | Frontal_Mid_R (aal) | 40 | 18 | 40 | 8 |
| 197 | FPN | Frontal_Mid_L (aal) | -34 | 55 | 4 |  |
| 198 | FPN | Frontal_Mid_Orb_L (aal) | -42 | 45 | -2 |  |
| 199 | FPN | Angular_R (aal) | 33 | -53 | 44 | 9 |
| 200 | FPN | Frontal_Mid_Orb_R (aal) | 43 | 49 | -2 |  |
| 201 | FPN | Frontal_Inf_Tri_L (aal) | -42 | 25 | 30 |  |
| 202 | FPN | Frontal_Sup_Medial_L (aal) | -3 | 26 | 44 |  |
| 203 | SAN | Cingulum_Mid_R (aal) | 11 | -39 | 50 |  |
| 204 | SAN | Parietal_Inf_R (aal) | 55 | -45 | 37 |  |
| 205 | SAN | Precentral_R (aal) | 42 | 0 | 47 |  |
| 206 | SAN | Frontal_Mid_R (aal) | 31 | 33 | 26 |  |
| 207 | SAN | Frontal_Inf_Tri_R (aal) | 48 | 22 | 10 |  |
| 208 | SAN | Insula_L (aal) | -35 | 20 | 0 |  |
| 209 | SAN | Insula_R (aal) | 36 | 22 | 3 |  |
| 210 | SAN | Frontal_Inf_Orb_R (aal) | 37 | 32 | -2 |  |
| 211 | SAN | Insula_R (aal) | 34 | 16 | -8 |  |
| 212 | SAN | undefined | -11 | 26 | 25 |  |
| 213 | SAN | Supp_Motor_Area_L (aal) | -1 | 15 | 44 |  |
| 214 | SAN | Frontal_Mid_L (aal) | -28 | 52 | 21 |  |
| 215 | SAN | Cingulum_Ant_L (aal) | 0 | 30 | 27 |  |
| 216 | SAN | Cingulum_Mid_R (aal) | 5 | 23 | 37 |  |
| 217 | SAN | Cingulum_Ant_R (aal) | 10 | 22 | 27 |  |
| 218 | SAN | Frontal_Mid_R (aal) | 31 | 56 | 14 |  |
| 219 | SAN | Frontal_Mid_R (aal) | 26 | 50 | 27 |  |
| 220 | SAN | Frontal_Mid_L (aal) | -39 | 51 | 17 |  |
| 221 | NL | Cingulum_Mid_R (aal) | 2 | -24 | 30 |  |
| 222 | subcortical | Thalamus_R (aal) | 6 | -24 | 0 |  |
| 223 | subcortical | Thalamus_L (aal) | -2 | -13 | 12 |  |
| 224 | subcortical | Thalamus_L (aal) | -10 | -18 | 7 |  |
| 225 | subcortical | Thalamus_R (aal) | 12 | -17 | 8 |  |
| 226 | subcortical | undefined | -5 | -28 | -4 |  |
| 227 | subcortical | Putamen_L (aal) | -22 | 7 | -5 |  |
| 228 | subcortical | undefined | -15 | 4 | 8 |  |
| 229 | subcortical | Putamen_R (aal) | 31 | -14 | 2 |  |
| 230 | subcortical | Putamen_R (aal) | 23 | 10 | 1 |  |
| 231 | subcortical | Putamen_R (aal) | 29 | 1 | 4 |  |
| 232 | subcortical | undefined | -31 | -11 | 0 |  |
| 233 | subcortical | undefined | 15 | 5 | 7 |  |
| 234 | subcortical | undefined | 9 | -4 | 6 |  |
| 235 | VAN | Temporal_Sup_R (aal) | 54 | -43 | 22 |  |
| 236 | VAN | Temporal_Mid_L (aal) | -56 | -50 | 10 |  |
| 237 | VAN | Temporal_Sup_L (aal) | -55 | -40 | 14 |  |
| 238 | VAN | Temporal_Sup_R (aal) | 52 | -33 | 8 |  |
| 239 | VAN | Temporal_Mid_R (aal) | 51 | -29 | -4 |  |
| 240 | VAN | Temporal_Sup_R (aal) | 56 | -46 | 11 | 7 |
| 241 | VAN | Frontal_Inf_Tri_R (aal) | 53 | 33 | 1 |  |
| 242 | VAN | Frontal_Inf_Tri_L (aal) | -49 | 25 | -1 |  |
| 243 | NL | Cerebelum_6_L (aal) | -16 | -65 | -20 |  |
| 244 | NL | Cerebelum_6_L (aal) | -32 | -55 | -25 |  |
| 245 | NL | Cerebelum_6_R (aal) | 22 | -58 | -23 |  |
| 246 | NL | Vermis_6 (aal) | 1 | -62 | -18 |  |
| 247 | NL | Fusiform_R (aal) | 33 | -12 | -34 | 2 |
| 248 | NL | Fusiform_L (aal) | -31 | -10 | -36 |  |
| 249 | NL | Temporal_Inf_R (aal) | 49 | -3 | -38 |  |
| 250 | NL | Temporal_Inf_L (aal) | -50 | -7 | -39 |  |
| 251 | DAN | Precuneus_R (aal) | 10 | -62 | 61 |  |
| 252 | DAN | Temporal_Mid_L (aal) | -52 | -63 | 5 |  |
| 253 | NL | Temporal_Inf_L (aal) | -47 | -51 | -21 |  |
| 254 | NL | Temporal_Inf_R (aal) | 46 | -47 | -17 |  |
| 255 | somato-motor | Postcentral_R (aal) | 47 | -30 | 49 |  |
| 256 | DAN | Parietal_Sup_R (aal) | 22 | -65 | 48 |  |
| 257 | DAN | Temporal_Mid_R (aal) | 46 | -59 | 4 |  |
| 258 | DAN | Parietal_Sup_R (aal) | 25 | -58 | 60 |  |
| 259 | DAN | Parietal_Inf_L (aal) | -33 | -46 | 47 |  |
| 260 | DAN | Occipital_Mid_L (aal) | -27 | -71 | 37 |  |
| 261 | DAN | Precentral_L (aal) | -32 | -1 | 54 |  |
| 262 | DAN | Temporal_Inf_L (aal) | -42 | -60 | -9 |  |
| 263 | DAN | Parietal_Sup_L (aal) | -17 | -59 | 64 |  |
| 264 | DAN | Precentral_R (aal) | 29 | -5 | 54 |  |
|  |  |  |  |  |  |  |
