# Supplementary material for: Does Aerobic Exercise Influence Intrinsic Brain Activity? An Aerobic Exercise Intervention among Healthy Old Adults
Source: Front Aging Neurosci. 2017 Aug 11;9:267. doi: 10.3389/fnagi.2017.00267 (PMC5554511; doi:10.3389/fnagi.2017.00267)
Supplement: Supplementary file 2 [file Table_2.DOCX]

**Supplemental Table S2**. *Correlations between cognition and aerobic capacity (VO2-peak).* Correlations at baseline (first column) and in change-change scores (second column). (Pearson correlation, p-value in parenthesis).

|  | Pre r(p) | Change r(p) |
| --- | --- | --- |
| Cog Score | 0.23 (0.12) | -0.09 (0.53) |
| Episodic Memory | -0.06 (0.67) | 0.00 (1.00) |
| Executive function | 0.25 (0.08) | -0.11 (0.46) |
| Processing speed | 0.31(0.03) | -0.18 (0.23) |
| Updating | 015(0.30) | 0.05(0.75) |
