# Supplementary figures and images for: Does Aerobic Exercise Influence Intrinsic Brain Activity? An Aerobic Exercise Intervention among Healthy Old Adults
Source: Front Aging Neurosci. 2017 Aug 11;9:267. doi: 10.3389/fnagi.2017.00267 (PMC5554511; doi:10.3389/fnagi.2017.00267)

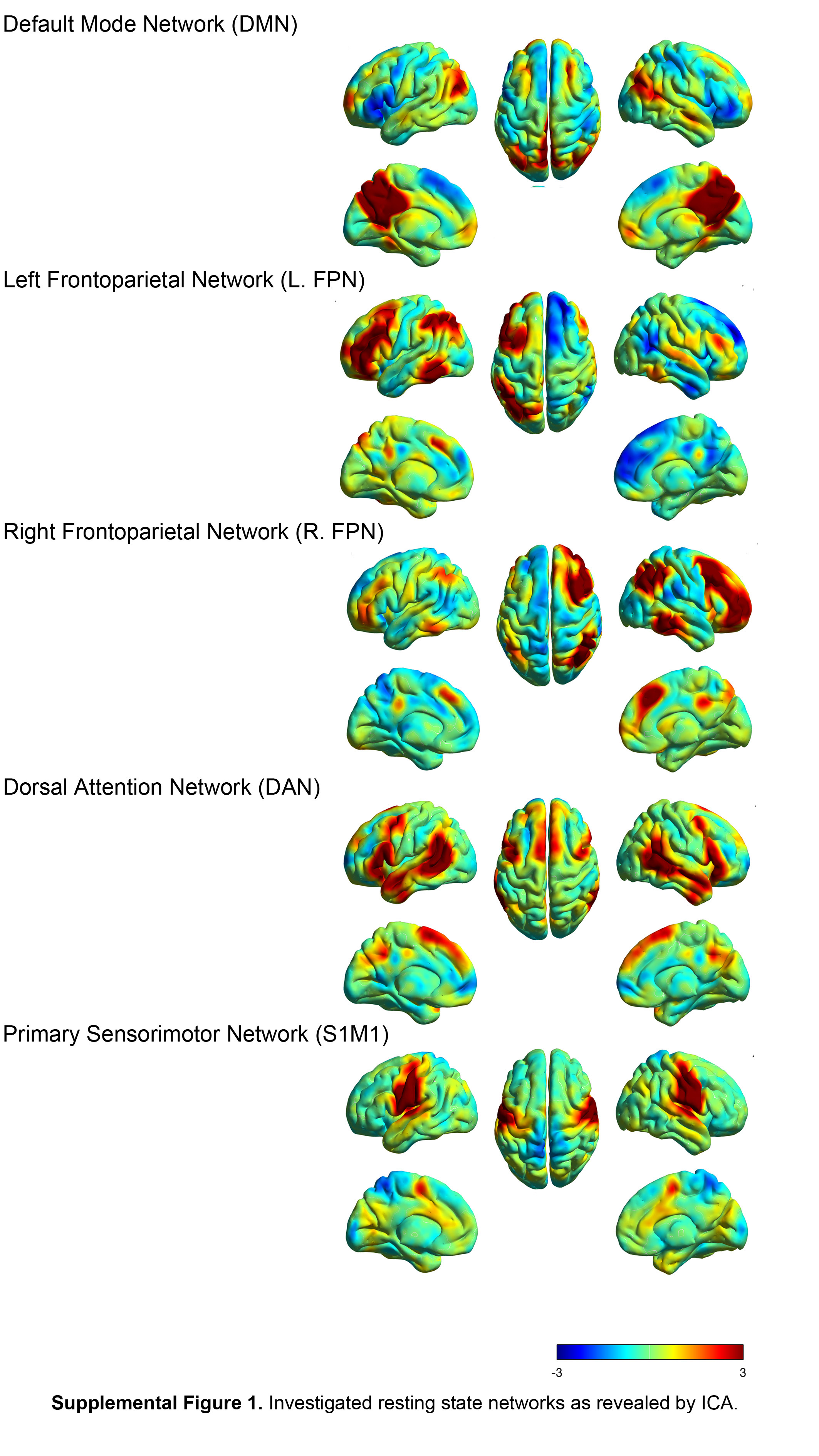

Supplement: Supplementary file 3 [file Image_1.JPEG]

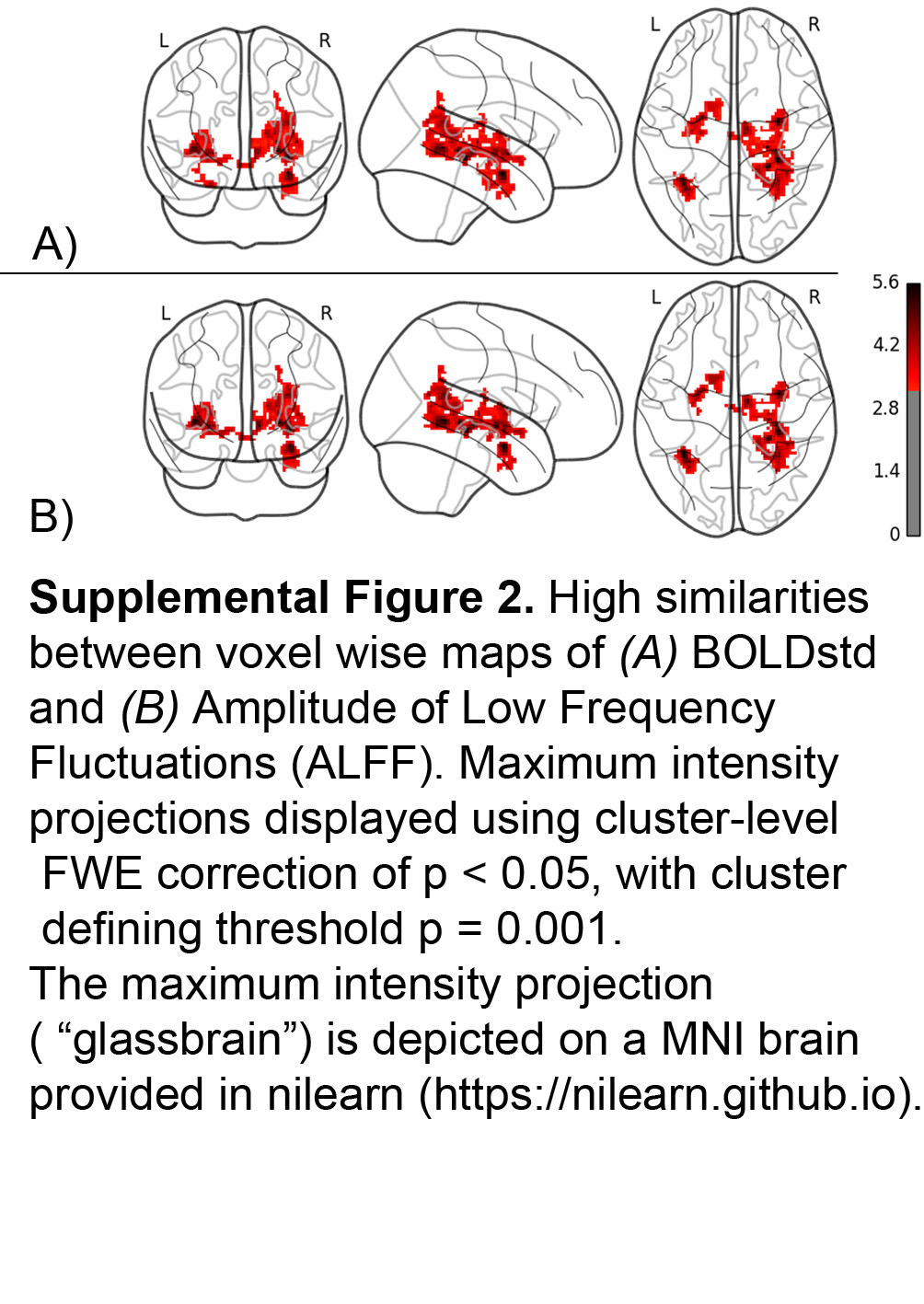

Supplement: Supplementary file 4 [file Image_2.JPEG]
